# Microwave ablation of malignant liver lesions: does the presence of cirrhosis affect the diameter of the ablation zones? A retrospective comparative study from a single institution

**DOI:** 10.1093/bjr/tqag154

**Published:** 2026-07-13

**Authors:** Behnam Shaygi, Rebecca Burger, Wyatt Reed, Nader Ashraf, Gina Weston-Petrides, Thomas Puttick, Asadi Hamed, Roberto Luigi Cazzato, Praveen Peddu

**Affiliations:** Northwick Park Hospital, Interventional Radiology, London, HA1 3UJ, United Kingdom; Imperial College School of Medicine, London, SW7 2AZ, United Kingdom; Imperial College Hospital NHS Trust, London, W2 1NY, United Kingdom; Marian University College of Osteopathic Medicine, Indianapolis, IN 45222-1997, United States; Imaging Institute, Cleveland Clinic, Cleveland, OH 44195, United States; King’s College Hospital NHS Foundation Trust, London, SE5 9RS, United Kingdom; Royal Surrey County Hospital, Guilford, GY2 7XX, United Kingdom; Monash Health, Melbourne, 3168, Australia; School of Medicine, Deakin University, Melbourne, 3125, Australia; The Florey Institute of Neuroscience and Mental Health, Parkville, Melbourne, Victoria 3052, Australia; University Hospital of Strasbourg, Strasbourg, 67091, France; King’s College Hospital NHS Foundation Trust, London, SE5 9RS, United Kingdom

**Keywords:** microwave ablation, oven effect, cirrhotic liver, hepatocellular carcinoma, liver metastases, thermal ablation

## Abstract

**Objectives:**

Thermal ablation techniques, such as radiofrequency ablation (RFA) and microwave ablation (MWA), are used to treat hepatic malignancies. The “oven effect,” where background cirrhotic tissue insulates and enhances heating, is well-documented in RFA but not in MWA. This study aims to explore whether the oven effect occurs in MWA.

**Methods:**

A retrospective comparative study included all patients undergoing MWA for hepatic malignancies between 2014 and 2017. Patients were divided into 2 groups based on liver lesion characteristics: H group (hepatocellular carcinoma, likely cirrhotic liver) and M group (metastases, likely non-cirrhotic liver). Ablation volumes were calculated using CT scans performed 6 weeks post-ablation. Multivariate linear regression analysis was performed to assess the significance of differences in ablation volume.

**Results:**

Ninety-four patients (57 in H group, 37 in M group) were included. Groups were demographically matched, and ablation parameters (intensity and duration) were comparable. The mean ablation volume in the M group was 2.3 cm^3^ smaller than in the H group, but this difference was not statistically significant (95% confidence interval −7.8, 3.3; *P* = .42).

**Conclusion:**

This study found no evidence of the oven effect phenomenon in MWA. This may result from MWA’s ability to achieve higher temperatures and larger ablation zones, reducing the impact of local tissue characteristics.

**Advances in knowledge:**

This study provides preliminary evidence that MWA produces consistent ablation volumes regardless of liver background, suggesting it is a reliable method for both cirrhotic and non-cirrhotic livers, advancing patient-specific treatment planning. However, prospective validation is required before integration into clinical guidelines.

## Introduction

Thermal ablation techniques utilize probes to create a cytotoxic thermal environment with the aim of destroying a specific area of tissue. They are increasingly popular procedures for the minimally invasive treatment of cancer within the liver.^1^ These techniques include radiofrequency ablation (RFA), microwave ablation (MWA), and cryoablation. These methods use thermal energy to create abnormally high or low temperatures in the targeted cancerous tissue, causing destruction.^2^ The standard of care has traditionally been RFA, but MWA has recently come into popularity because of the ability to reach higher temperatures and larger ablation zones in less time.^3^

For a thermal ablation technique to be effective, it needs to deliver a significant thermal effect in a targeted tissue, with maximal effect on that tissue only and minimal collateral damage to surrounding tissue. The degree to which this occurs is in part dependent on the nature of the surrounding tissue itself—which is the question posed by our research. Studies in RFA have demonstrated the phenomena described as the “oven effect” and “heat sink.”^4^^,^ ^5^ Heat sink describes the dissipation of heat via adjacent vascular structures that create a constant flow of blood “carrying” heat away from the target. The oven effect describes an environment where heat sink is minimized by the lack of surrounding vasculature and presence of increased fibrosis, so that the tissue target is in effect “insulated” thereby increasing the heat to that tissue target for the same delivered energy. Cirrhotic liver, which is less vascularized, is expected to suffer less from heat sink and provide an insulating layer, resulting in increased heating and larger ablation zone.^4^ There is now mounting supportive evidence that the nature of the tissue surrounding the lesion influences the efficacy of ablation.^4^^,^ ^6^

The oven effect has been most frequently studied in hepatocellular carcinoma (HCC), as this nearly always occurs in patients with background cirrhosis. In comparison, hepatic metastases tend to occur in healthy background liver and therefore provides a suitable comparison.^7^^,^ ^8^ In contrast to RFA, MWA utilizes higher temperatures delivered over a shorter period. This results in reduced procedure times, larger and more spherical ablation zones, and higher achieved temperatures.^9^ Though both ablative procedures appear similar in methods, results can vary significantly.^10^ While the oven effect is now well-documented in RFA, it has not been thoroughly explored in relation to MWA.

The aim of this study is to explore whether the oven effect phenomenon also occurs with MWA, with the aspiration that the consequences of this observation will lead to patient benefit and future patient-specific device protocols. Our goal is to further optimize ablative methods to provide a less invasive, lower cost treatment with quicker recovery time and fewer complications compared to current standard surgical practices. Understanding the relevance of background liver tissue in selection of the ablation method will allow us to tailor treatments to individual patient groups and move toward creating further evidence-based treatment guidelines.

## Methods

We performed a retrospective comparative study. All patients who underwent MWA for malignant hepatic lesions at a single institution, between January 2014 and December 2017 were considered for analysis. We included all the patients who had undergone MWA of a hepatic lesion, using a single cycle and Solero MWA system. As this study was retrospective at not involving patient identifiable information, ethical approval was not required.

We reviewed the electronic records for each patient and recorded patient-specific data, primary diagnosis, applied energy, and ablation duration. We reviewed the first follow-up CT scan, scheduled to occur 6 weeks post procedure, and from this scan calculated ablation volumes. Volume of the ablation area was calculated using ellipsoid volume formula ([4π/3] × height/2 × length/2 × width/2). Three-dimensional measurements of the target area were acquired from multiplanar reformat (MPR) images (Figure 1). Ellipsoid volume formula was used to approximate area as segmentation software was not available in our institution. All ablation measurements were performed by a single reader (TP). In cases where there was uncertainty regarding measurement accuracy (a small minority of cases), images were reviewed jointly with a senior author (BS) to reach a consensus.

**Figure 1:**
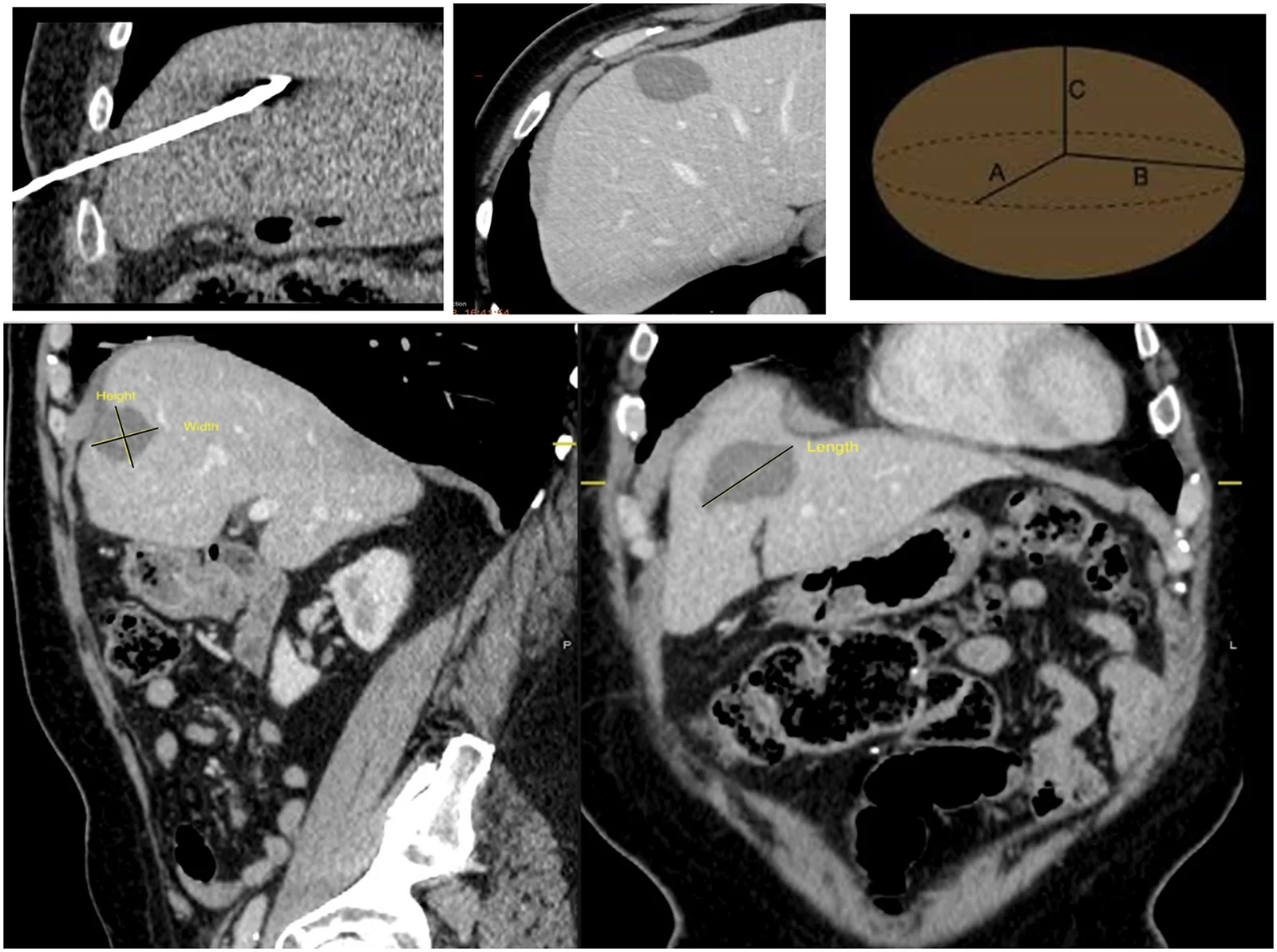
MPR images used to calculate volume of the ablation area. Ablation area volumes were calculated using ellipsoid approximation.

Imaging performed 6 weeks post procedure was used to allow for resolution of post therapeutic hyperemia and necrosis.

Patients were divided into groups based on the characteristics of their liver lesion and background liver disease. They were divided into either the H group (ablation performed for HCC, and therefore likely to have liver cirrhosis) or M group (ablation performed for hepatic metastasis, and therefore likely to have healthy liver tissue) (Figure 2).

**Figure 2:**
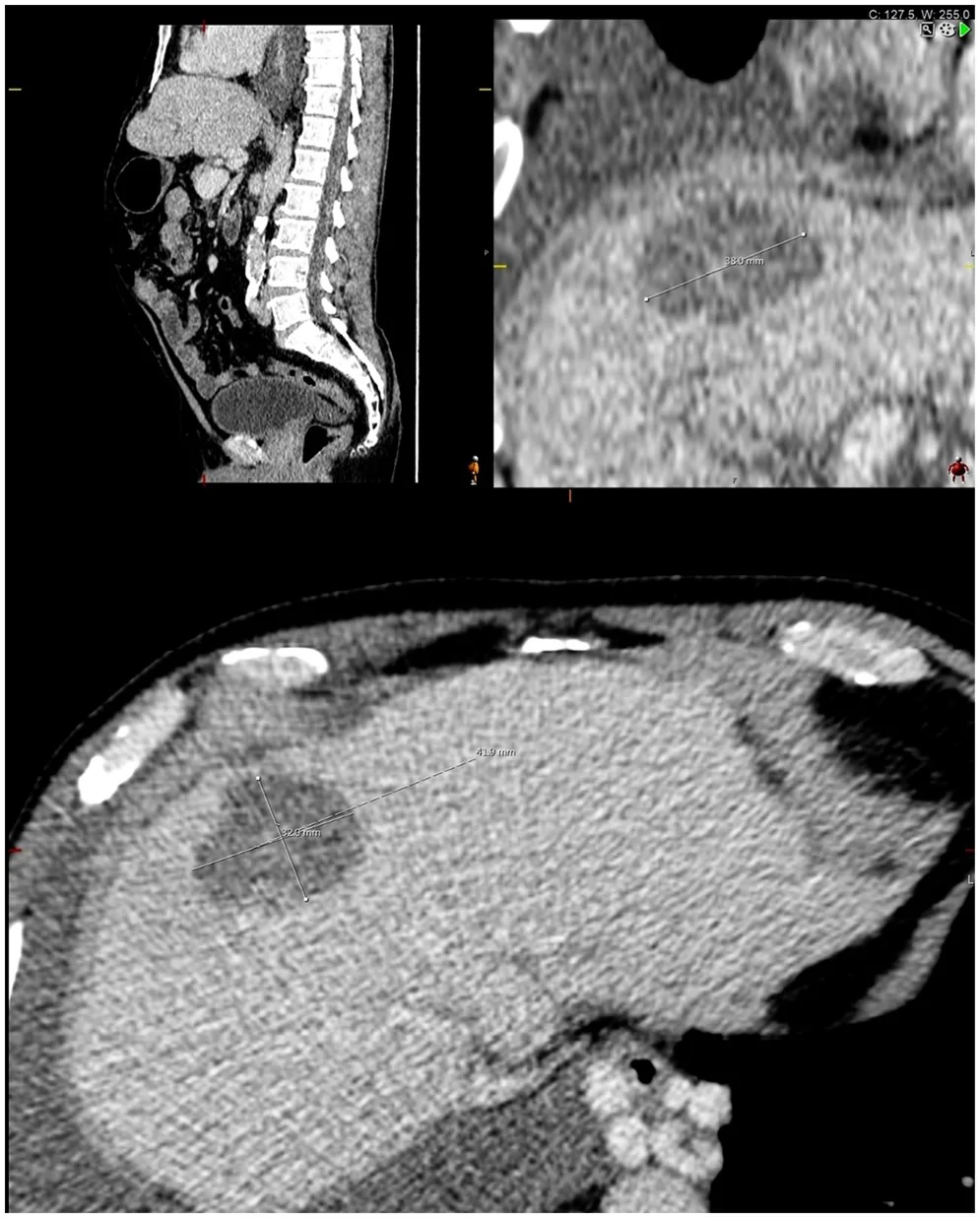
Sagittal, coronal, and axial sequences demonstrating post ablation appearances in a cirrhotic liver.

Patients were excluded from the study if: follow-up imaging was not available, multiple ablation cycles were performed for a single lesion, the lesion represented a repeat (previously ablated) procedure.

### Statistical analysis

Continuous variables were calculated using Microsoft Excel (Microsoft Office, 2016). To assess for any significant variation between the variables we conducted a multivariate linear regression analysis using Statistical Package for the Social Sciences (SPSS, IBM Corp, Armonk, NY, United States), adjusting for age, sex, power and duration of ablation in regard to ablation volume. A *P-*value <.05 was considered significant.

### Procedure

The ablation procedure was performed by multiple operators, with a standardized protocol to reduce operator variability. The procedure was performed under general anesthesia with the Solero microwave tissue ablation system (Solero MTA; AngioDynamics, Latham, NY, United States). The procedure was guided by computed tomography with a 40-channel scanner (Siemens Somatom Definition 128 Slice) and by ultrasound (Logiq E9; GE Healthcare, Chicago, IL, United States). Following prepping and draping the area and under image guidance, the microwave antenna was introduced into the liver lesion by percutaneous insertion, and microwave power was applied in accordance with the lesion size and location. At the end of the procedure, after repeat imaging in CT, the ablated area was deemed to be satisfactory in size and the patient was transferred to recovery.

## Results

A total of 123 consecutive cases undergoing microwave ablation were retrospectively identified. 29 patients were excluded, and a total of 94 patients (69 male and 25 female) were included in the final analysis (Figure 3). Mean (standard deviation [SD]) age of the participants was 65.3 (11.2) years. There was no statistically significant difference in mean age (*P-*value .72) between the 2 groups. 57 patients were treated for HCC and 37 for cancer metastasis (25 were metastasis from colorectal cancer, 5 from pancreatic cancer, 1 from breast cancer and 1 from choriocarcinoma).

**Figure 3:**
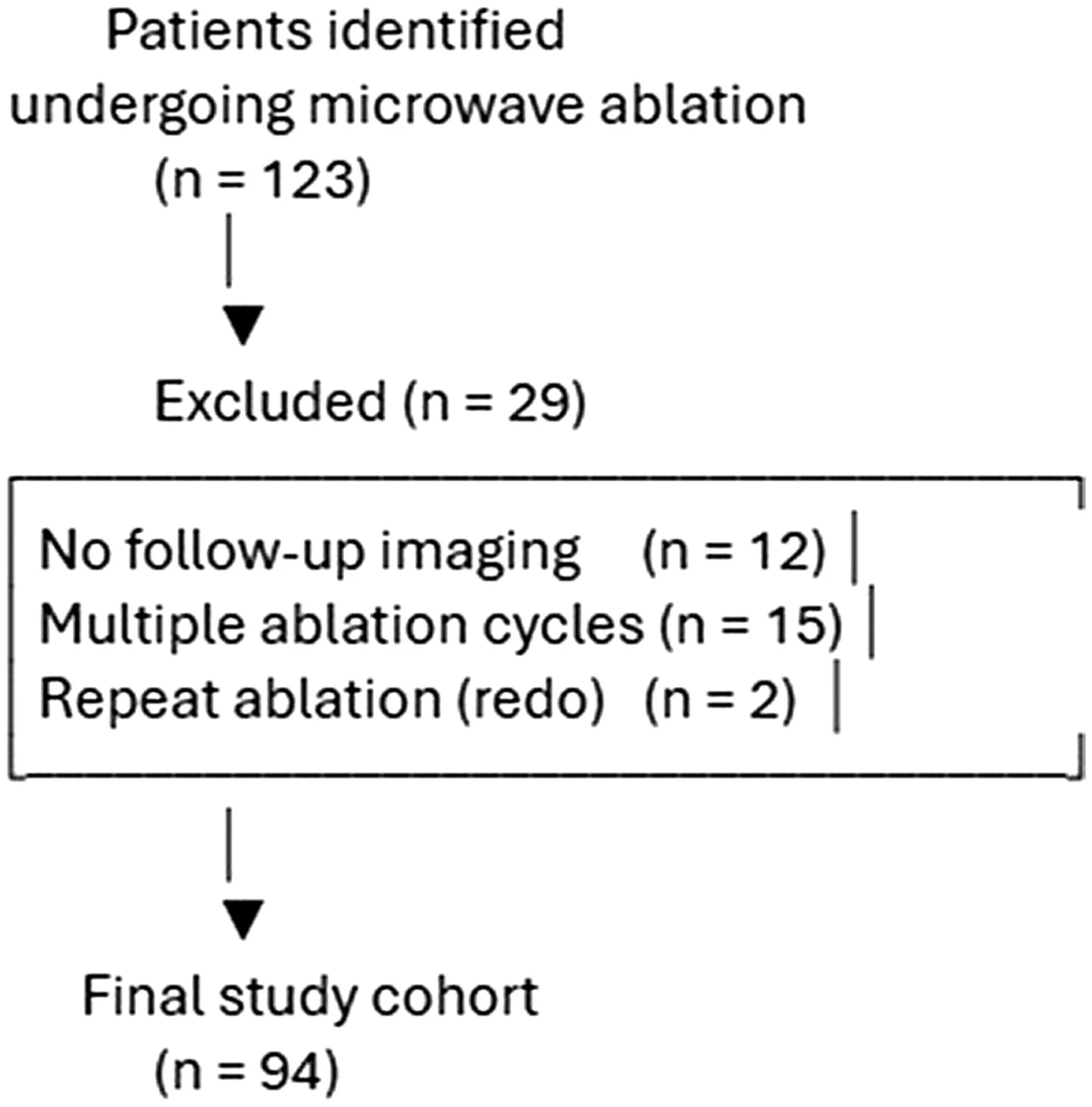
Patient flow diagram detailing study selection process. A total of 29 patients were excluded from the study, and 94 included in the final analysis.

The overall mean (SD) of required power (in watts) and duration (in minutes) was 120 (21.4) watts and 4.8 (1.1) minutes, respectively, with no difference between the 2 groups. The mean (SD) of required power (in watts) for H group was 119.3 (19.6) and M group 121.1 (24), *P-*value .69. The mean (SD) for duration in minutes for H group was 4.7 (1.03) and M group 4.9 (1.2), *P-*value .39.

Mean (SD) volume of the ablation was 21.3 cm^3^ (14.1) in the H and 19.5 cm^3^ (13.8) in the M group.

In linear regression analysis adjusted for age, sex, power, and duration of ablation, the ablation volume in the metastasis group was smaller than the HCC group. However, this difference was not statistically significant (mean difference −2.3 cm^3^; 95% confidence interval −7.8, 3.3). The ablation volume was smaller in women compared to men, but this difference did not reach statistical significance. As expected, a higher power in Watts was associated with larger ablation volume (mean difference in ablation volume per 10 watts higher power was 2.4; 95% confidence interval 1.1, 3.7), and this difference was statistically significant (*P* < .001) (Table 1, Figure 4).

**Figure 4:**
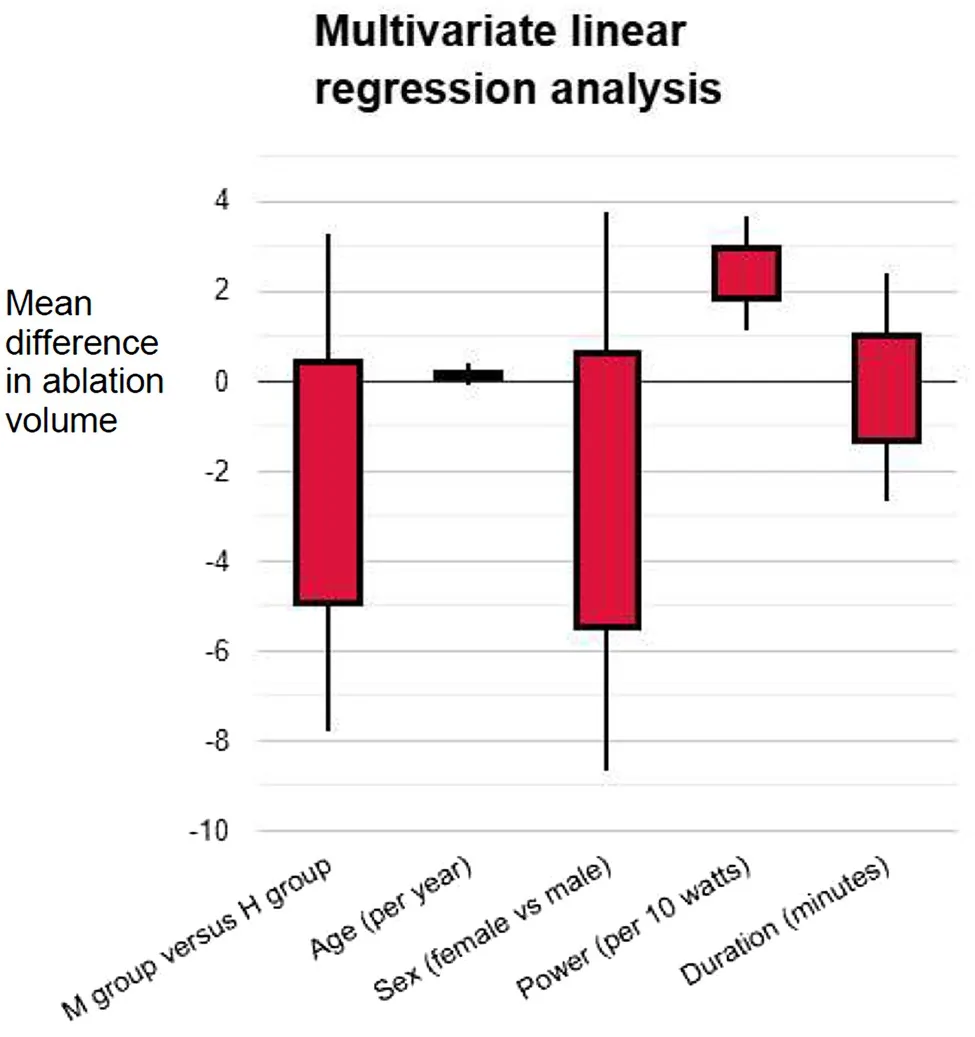
Graphical representation of Table 1. Findings of the multivariate linear regression analysis, demonstrating the effect of the variables on ablation volume. Mean difference in ablation volume and 95% confidence intervals demonstrated.

**Table 1:** Multivariable linear regression analysis adjusted for age, sex, power, and duration of ablation.

Volume	Mean difference (95% confidence interval)	*P*-value
**Metastasis group compared to HCC group**	−2.3 (−7.8, 3.3)	.42
**Age (per year)**	0.2 (−0.1, 0.4)	.12
**Sex (female vs male)**	−2.4 (−8.7, 3.8)	.44
**Power (per 10 watts)**	2.4 (1.1, 3.7)	<.001
**Duration (per minutes)**	−0.2 (−2.7, 2.4)	.89

## Discussion

This study demonstrated that when adjusted for age, intensity, and duration of ablation, there was no statistically significant difference in ablation volume between the cirrhotic and non-cirrhotic liver groups. Although the mean ablation volume in the non-cirrhotic liver group was 2.3 cm^3^ smaller than the cirrhotic liver group, the difference was not significant (95% confidence interval −7.8, 3.3) and we therefore did not find a difference between the groups. While the results of this pilot study are consistent with previous literature on the heating characteristics of MWA,^4^^,^ ^11^ the results of our study do not demonstrate an oven effect relationship in MWA.

We hypothesize that a potential explanation for the lack of oven effect in MWA is due to the unique properties of MWA, which allow for larger ablation zones and higher temperatures in a shorter period compared to other modalities, minimizing the effect of local tissue characteristics.^12^^,^ ^13^

However, another explanation for our results is the small sample size meant that our study did not have sufficient power to find a difference between the groups and is a significant limitation of our study. Additionally, a potential confounder could be tumor location, as the “heat sink” effect of adjacent tissue structures may also affect heating characteristics.^14^ Furthermore, all procedures were performed with a single type of device; comparing different devices may provide more insight and provide information if device manufacturing contributes to potential oven effect. Further research on the relationship between MWA and the oven effect would greatly benefit from larger samples.

Moreover, due to various enhancement patterns of different types of lesions, only the post-ablation follow-up images were used for calculation and comparison of the ablation zone volumes. However, this potential confounder was addressed by analyzing the volumes in the cases whereby only one ablation was used for treatment according to the manufacturer protocol and the energy and duration of the ablation cycles for both groups were comparable and did not show any statistically significant difference. The timing of post-ablation assessment highlights a further key compromise, as delaying evaluation until 6 weeks post procedure will reduce the confounding effects of post treatment hyperemia over-estimating residual tumor volume. However, the delayed time point may also allow for interval tumor progression. The 6 week time point was chosen as a pragmatic balance between the 2 considerations, and is established practice elsewhere.^15^

Another limitation of our study was that we relied on the assumption that patients with hepatocellular carcinoma have underlying cirrhosis, while patients with metastatic disease have healthy background liver tissue. While nearly all patients with hepatocellular carcinoma do have underlying cirrhosis,^7^ our characterization was not based on histological diagnosis.

An advantage of this study is that we were able to control for age, intensity, and duration while maintaining similar study sizes between groups. Obtaining comparable sample sizes is often difficult due to the prevalence of HCC compared to liver metastases.^16^

The concept of the oven effect was initially demonstrated by Liu et al. However, research following this paper has provided inconsistent conclusions on the true relevance of the oven effect on radiofrequency ablation procedures. While many articles do suggest some oven effect,^4^^,^ ^17^ there are also contradictions. A study on the oven effect utilizing MWA expands on the literature by evaluating an ablative modality that differs from RFA and identifies if the oven effect is extendable to other modalities.

Understanding further characteristics of MWA, oven effect, and lack of relationship provides insight to the clinical decision making when undergoing these procedures. This pilot study aids in the information surrounding when and why RFA or MWA could benefit patients depending on the nature of the lesion in the liver. The study provides preliminary evidence that MWA is equally efficacious in patients with and without cirrhosis, and suggests MWA can be a viable option for patients regardless of liver background. Physicians can ensure that when providing MWA for patients who have primary liver cancer or metastases, their liver background does not confound the predictability of the dimensions of the ablation area. As MWA continues to be more popular as an ablation choice, this study adds to the evidence that MWA is a reliable method regardless of background liver characteristics. However, further prospective research addressing the limitations in sample size and fibrosis quantification should be undertaken before integration into clinical guidelines.

According to our study, there was no statistically significant difference in ablation volumes post MWA, regardless of the background liver disease in cirrhotic or healthy livers.
